# The green tea catechin epigallocatechin gallate inhibits SARS-CoV-2 infection

**DOI:** 10.1099/jgv.0.001574

**Published:** 2021-04-08

**Authors:** Lisa Henss, Arne Auste, Christoph Schürmann, Christin Schmidt, Christine von Rhein, Michael D. Mühlebach, Barbara S. Schnierle

**Affiliations:** ^1^​ Department of Virology, Paul-Ehrlich-Institut, Paul-Ehrlich Strasse 51-59, 63225 Langen, Germany; ^2^​ Department of Veterinary Medicine, Paul-Ehrlich-Institut, Paul-Ehrlich Strasse 51-59, 63225 Langen, Germany; ^3^​ German Center for Infection Research, Gießen-Marburg-Langen, Germany

**Keywords:** Green tea, EGCG, SARS-CoV-2, pseudotype, MERS-CoV, SARS-CoV

## Abstract

The severe acute respiratory syndrome coronavirus-2 (SARS-CoV-2) infection has caused a pandemic with tens of millions of cases and more than a million deaths. The infection causes COVID-19, a disease of the respiratory system of divergent severity. No treatment exists. Epigallocatechin-3-gallate (EGCG), the major component of green tea, has several beneficial properties, including antiviral activities. Therefore, we examined whether EGCG has antiviral activity against SARS-CoV-2. EGCG blocked not only the entry of SARS-CoV-2, but also MERS- and SARS-CoV pseudotyped lentiviral vectors and inhibited virus infections *in vitro*. Mechanistically, inhibition of the SARS-CoV-2 spike–receptor interaction was observed. Thus, EGCG might be suitable for use as a lead structure to develop more effective anti-COVID-19 drugs.

## Introduction

The major constituent and most important polyphenolic catechin in green tea is epigallocatechin-3-gallate (EGCG). Other catechins are also found in green tea extract, such as epigallocatechin (EGC), epicatechin gallate (ECG) and epicatechin (EC). They all have a basic flavan-3-ol structure, but EC does not contain a galloyl side chain, which is thought to contribute to the biological activities of EGCG [1]. EGCG has been described to have antiviral activities towards a variety of viruses, although the exact mechanism of these inhibitory effects is not yet understood. It inhibits the cell entry of several viruses, such as human immunodeficiency virus (HIV) [2–4], influenza virus [5], hepatitis C virus (HCV) [6–9], and chikungunya virus [10]. On the other hand, inhibitory effects on viral transcription have been reported for viruses like hepatitis B virus (HBV), adenoviruses, or herpesviruses. For a review, see [11, 12].

The severe acute respiratory syndrome coronavirus-2 (SARS-CoV-2) belongs to the *Coronaviridae* family and is the causative agent of mainly pneumonias, defined as Corona Virus Disease-2019 (COVID-19), which first emerged in the Hubei province in China [13]. After only about 4 months, the virus had spread worldwide and the SARS-CoV-2 pandemic was declared in March 2020 by the World Health Organization. SARS-CoV-2 infection can cause severe life-threatening disease with high mortality rates in older patients and patients with comorbidities [14, 15].

The Coronaviruses are enveloped positive-strand RNA viruses with large genomes. They cause disease in diverse animal species and in humans. The human coronaviruses hCoV-OC43, hCoV-229E, hCoV-HKU1, and hCoV-NL63 are the causative agents of common colds. However, the severe acute respiratory syndrome virus (SARS-CoV) and the Middle East respiratory syndrome virus (MERS-CoV) have a high pathogenic potential with 10–30% lethality in humans [16].

Currently, with the exception of remdesivir, there is no approved, specific treatment of the SARS-CoV-2 infection available. Traditional medicine and plant extracts are currently screened for their antiviral activities and for instance the antimalarial drug artemisinin, produced from the medicinal plant, *Artemisia annua* L., has been described to have anti-SARS-CoV-2 activity *in vitro*. In China, 85 % of SARS-CoV-2 infected patients have received in addition to conventional therapies also Traditional Chinese Medicine (TCM) treatment and current knowledge was recently summarized [17–19].

Therefore, we were interested in the influences of EGCG on SARS-CoV-2 infection.

## Methods

### Cell culture

HEK293T-hACE2 [20] cells were cultured at 37 °C under 5 % CO_2_ and grown in Dulbecco’s modified Eagle medium (DMEM; Sigma-Aldrich, Taufkirchen, Germany) supplemented with 10 % foetal bovine serum (Sigma-Aldrich, Taufkirchen, Germany), 5 % l-glutamine (200 mM; Lonza, Verviers, Belgium), and 1 % penicillin/streptavidin (Fisher Scientific, Schwerte, Germany). Vero cells (ATCC CCL-81) and Huh7 cells (CSC-C9441L) were cultured in DMEM supplemented with 10 % foetal bovine serum and 1 % l-glutamine. SARS-CoV-2 (isolate MUC-IMB-1) was a kind gift of G. Dobler, Bundeswehr Institute for Microbiology, Germany. MERS-CoV strain EMC/2012 was provided by Ron Fouchier (Erasmus University, Rotterdam, The Netherlands [21];) and SARS-CoV strain Frankfurt-1 by Christian Drosten (Institute of Virology, Charite, Berlin, Germany [22]). Epigallocatechin gallate (EGCG) and epicatechin (EC) were purchased from Sigma-Aldrich (Taufkirchen, Germany).

### Pseudotype-based neutralization assay

Lentiviral vectors were prepared in HEK293T cells by co-transfection using Lipofectamine 2000 (Thermo Fisher, Darmstadt, Germany), as described previously [23]. Plasmids encoding HIV-1 gag/pol, rev, and the luciferase-encoding lentiviral vector genome (pMDLg/pRRE, pRSVrev, pRRLsinCMV-GFPpre [18], pCSII-Luc [19]) were transfected together with the SARS-CoV-2 delta 19 spike gene cloned into the plasmid pcDNA (Genbank #MN908947; synthesized by Eurofins, Ebersberg, Germany). In addition, other spike encoding plasmids were used: The SARS-CoV spike gene in pcDNA (#AY278741.1, codon-optimized); the plasmid pGAGGS-MERS-CoV-S encoding a codon-optimized MERS-S gene (AFY13307.1; generous gift of Nigel Temperton, University of Kent, UK [24]), the codon-optimized NL63 delta 19 spike gene (#AFV53148.1) cloned into the plasmid pcDNA [25] or the pHIT-G plasmid encoding the VSV-G gene [26]. Cell culture supernatants containing the vectors were concentrated by ultracentrifugation for 1 h at 50 000 r.p.m. and stored at –80 °C. Pseudotyped vectors and serially diluted EGCG or EC (20–0.625 µg ml^−1^) were incubated in triplicate for 30 min at 37 °C and used to transduce 6000 HEK293T-hACE2 cells in 384-well plates. After 24 and 48 h, the luciferase substrate BriteLite (PerkinElmer, Rodgau, Germany) was added to measure luciferase activities. The 50 % inhibitory value (IC_50_) calculated for each sample corresponds to the neutralization activity. IC_50_ was calculated using the GraphPad Prism 7.04 software (La Jolla, CA, USA).

### Plaque reduction assay

The green tea compounds were two-fold serially diluted in DMEM supplemented with 2 mM l-glutamine in duplicate and mixed with 50 µl medium containing 1×10^2^ TCID_50_ SARS-CoV-2 (isolate MUC-IMB-1) in a total volume of 100 µl. The virus–compound mixture was incubated at 37 °C for 30 min, added to 8×10^5^ Vero cells that had been seeded in 6-well plates the day before, and then incubated for 1 h at 37 °C. After removal of the inoculum, cells were overlaid with DMEM supplemented with 2 mM l-glutamine, 1.5 % microcrystalline cellulose Avicel RC-591NF (FMC BioPolymer, Co. Cork, Ireland), and 2 % foetal bovine serum and EGCG or EC of the indicated concentrations. After 3 days, the overlay medium was removed and the cells were fixed with 4 % formalin in PBS and then stained with crystal violet to visualize plaques in the confluent cell monolayer. The plaques were counted and the IC_50_ was calculated using the GraphPad Prism 7.04 software (La Jolla, CA, USA).

### Surrogate neutralization test (sVNT)

The SARS-CoV-2 sVNT Kit (Genscript, Leiden, Netherlands) is a blocking ELISA that mimics the virus receptor-binding process. Inhibition of the protein–protein interaction between a horseradish peroxidase (HRP)-conjugated recombinant SARS-CoV-2 spike receptor-binding domain fragment (HRP-RBD) and hACE2 is measured as a surrogate for neutralization. The assay was performed with different concentrations of EGCG or EC following the manufacturer’s instructions. The negative (non-inhibiting) and positive (inhibiting) controls of the sVNT kit were included. Inhibition was calculated following the manufacturer’s protocol using the following equation: Inhibition in % = (1 – OD of sample/OD of negative control)×100. The cutoff was set to 0, the value of the negative control.

### Cell toxicity assay

Toxicity of the compounds was determined by using the ATPlite 1step Luminescence Assay System of Perkin Elmer (Rodgau, Germany). Cells were incubated with EGCG or EC at different dilutions for 24 or 48 h in triplicate and analysed by adding 7.5 µl ATPlite-substrate per well, followed by detection of luciferase units with PHERAstar (BMG LABTECH, Ortenberg, Germany). The viability data are given as % relative light units of solvent (DMEM)-treated cells.

### Statistical analysis

Statistical analyses were performed using the GraphPad Prism 7.04 software (La Jolla, CA, USA). IC_50_ and CC_50_ values were calculated as nonlinear regression: Log (inhibitor) vs response (three parameter) constrain equal to 0. *P*-values were calculated as column analyses: Student’s *t*-test (and nonparametric test) unpaired.

### Time-of-drug-addition assay

Vero cells (2×10^4^), which had been seeded into 96-wells the day before, were infected with SARS-CoV-2 (isolate MUC-IMB-1, MOI=1.5). After 1 h of incubation at 37 °C, cells were washed once with ice-cold PBS and fresh DMEM supplemented with 2 mM l-glutamine and 10 % foetal bovine serum was added. Then 1 h before start of infection, 1 h or 6 h after the start of the infection, medium was changed either with medium containing 10 µg ml^−1^ EGCG or medium only. After 11 h post-infection, cells were lysed and analysed using RT-qPCR. The RNA was isolated from infected Vero cells using TRIzol Reagent (Ambion, Thermo Fisher Scientific, Dreieich Germany) and Direct-zol RNA MiniPrep kit (Zymo research, Freiburg im Breisgau, Germany) according to manufacturer’s instructions. RNA was resuspended in 50 µl RNase-free water. SARS-CoV-2 RNA was quantified using Superscript III one step RT-PCR system with Platinum Taq Polymerase (Invitrogen, Darmstadt, Germany) detecting the E gene as described by Corman *et al*. [27]. Reactions were performed in 96-wells with 5 µl of RNA in a total volume of 25 µl and run in triplicates on a CFX 96 qPCR cycler (Bio-Rad Laboratories, Hercules, CA). For analysis of samples, RNA of an SARS-CoV-2 infected hamster was used as internal standard, which had been validated with reference standard (NIBSC 19/304) before (linear range, 4.5×10^6^ to 4.5×10^2^ copies [28]). The cycling conditions were as follows: reverse transcription for 10 min at 55 °C, followed by denaturation for 3 min at 94 °C, and 45 cycles of 15 s at 94 °C and 30 s at 58 °C. Quantified sample copy numbers were normalized to cell count and are presented as mean – standard error of three independent experiments.

## Results

### EGCG inhibits pseudotyped vectors and has broad antiviral activity

The first steps of infection by SARS-CoV-2 are mediated by its spike glycoprotein, which consists of two subunits, S1 and S2. S1 facilitates the attachment of the virus to cells via its receptor-binding domain (RBD), and the S2 domain mediates the fusion of viral and cellular membranes. SARS-CoV-2 utilizes the host protein, angiotensin-converting enzyme 2 (ACE2) for binding and entry into human cells. In addition, proteolytic cleavage of S1 by the serine protease TMPRSS2 is required [29]. Antibodies that bind to the spike RBD can neutralize SARS-CoV-2 [30, 31].

**Fig. 1.**
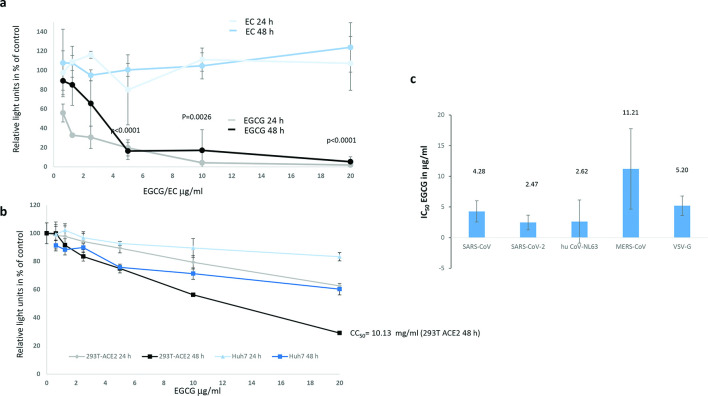
EGCG inhibits transduction with SARS-CoV-2-pseudotyped lentiviral vectors. (**a**) EGCG or EC were added at the indicated concentrations to SARS-CoV-2-pseudotyped vectors encoding luciferase, incubated for 30 min at 37 °C and then added to HEK293T-ACE2 cells. Gene transfer into HEK293T-ACE2 cells was analysed after 24 and 48 h by measurement of luciferase activity indicated as % of the untreated control. The values for EGCG are depicted in black and grey, and those for EC in blue and light blue. The 24 h incubation was done in triplicate. The values for the 48 h incubation are mean values of three experiments done in triplicate. Significant differences between the untreated control and EGCG treatment for 48 h at the indicated concentrations are shown by the *p*-values, which were calculated using Student’s *t*-test. The -values for EGCG concentrations lower than 5 µg ml^−1^ were not significant. (**b**) Toxicity of EGCG and EC on HEK293T-ACE2 and Huh7 cells was tested by monitoring ATP with a luminescence assay. Cell viability (as relative light units) after 24 h incubation with EGCG is depicted in grey and viability after 48 h in black for 293T-ACE2 cells and in dark blue and light blue for Huh7 cells. The values for both assays are mean values of three experiments done in triplicate (Huh7 cells: one experiment in triplicate). (**c**) Gene transfer by SARS-CoV-, NL63-,and VSV-G-pseudotyped vectors into HEK293T-ACE2 cells and MERS-CoV vectors into Huh7 cells, was analysed in triplicate after 48 h by measurement of luciferase activity in five independent experiments. The corresponding mean IC_50_ values and their standard deviations are depicted and were calculated using the GraphPad Prism 7.04 software (La Jolla, CA, USA).

**Fig. 2.**
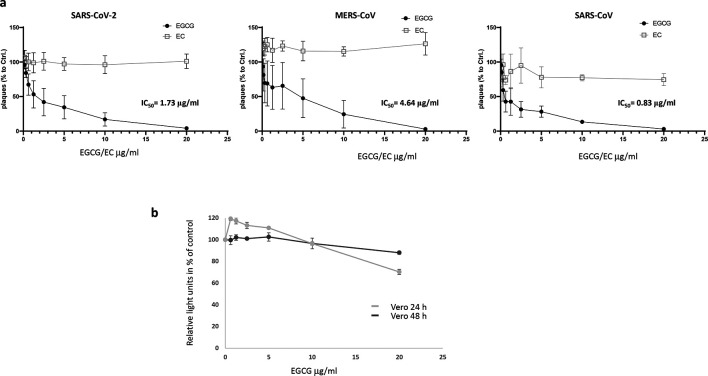
EGCG inhibits coronavirus infections. (**a**) Vero cells were infected with SARS-CoV-2, MERS- or SARS-CoV in the presence of EGCG or EC. The values are mean values of three experiments (MERS-CoV *n*=4) performed in duplicate. Significant differences between EC and EGCG treatments are indicated as *p*-values 0.0009 for SARS-CoV-2,<0.0001 for MERS-CoV and 0.0023 for SARS-CoV. *P*-values were calculated with Student’s *t*-test, using the GraphPad Prism 7.04 software (La Jolla, CA, USA). (**b**) Toxicity of EGCG and EC on Vero cells was tested by monitoring ATP with a luminescence assay. Cell viability (as relative light units) after 24 h incubation with EGCG is depicted in grey and viability after 48 h in black. The values for both assays are mean values of two experiments done in triplicate.

To determine if EGCG blocks SARS-CoV-2 cell entry, we transduced HEK293T-ACE2 cells [20] with SARS-CoV-2 spike protein-pseudotyped lentiviral vectors encoding luciferase in the presence of different amounts of EGCG or EC. Pseudotyping of lentiviral vectors with the SARS-CoV-2 protein is a useful tool to study SARS-CoV-2 entry at a low biosafety level [32]. Inhibition of vector entry is indicated by a reduced luciferase activity of the transduced cells [25]. A clear, dose-dependent inhibition of SARS-CoV-2-pseudotyped vectors by EGCG was observed after 24 and 48 h incubation, while EC had no effect (Fig. 1a). In addition, potential toxicity of EGCG and EC was tested by monitoring ATP with a luminescence assay. Cell viability after 24 h incubation with EGCG, as relative light units in percent of untreated control, is depicted in grey and viability after 48 h in black (Fig. 1b). As indicated in Fig. 1b, only high concentrations of EGCG reduced cell viability after 48 h incubation. Inhibition of SARS-CoV-2-pseudotyped vector entry is specific, because 5 µg ml^−1^ EGCG hardly affected cell viability, but reduced the transduction rate to 16 % compared to the untreated control. EC had no effect on cell viability (data not shown). The IC_50_ values for the inhibition of SARS-CoV-2-pseudotyped vectors were 2.47 µg ml^−1^ for EGCG and >20 µg ml^−1^ for EC, suggesting that EGCG interferes with SARS-CoV-2 entry processes while EC is ineffective. Similarly, EGCG interference with vectors pseudotyped with VSV-G and the receptor-binding proteins of SARS-CoV and the common cold human coronavirus NL63 or MERS-CoV were tested and the IC_50_ values were calculated as before (Fig. 1c). In contrast to the other coronaviruses tested, MERS-CoV does not use ACE2 as a receptor and vector inhibition was tested in Huh7 cells. Huh7 cell viability was not affected by EGCG or EC (CC_50_ >20 µg ml^−1^; Fig. 2b). EGCG treatment inhibited the ACE2 receptor using coronavirus-pseudotyped vectors with IC_50_ values of 4.28 µg ml^−1^ EGCG for SARS-CoV and 2.62 µg ml^−1^ for NL63. MERS-CoV and the VSV-G control were inhibited to a lesser extent (11.21 and 5.20 µg ml^−1^ EGCG respectively); however, a broad antiviral activity of EGCG was confirmed (Fig. 1c).

### EGCG inhibits SARS-CoV-2 virus replication

To validate the potential effect of EGCG, we tested its influence on the infection of Vero cells with three emerging pathogenic coronaviruses: SARS-CoV-2, MERS- and SARS-CoV [33–35]. The green tea compounds EGCG and EC were two-fold serially diluted in DMEM and preincubated at 37 °C for 30 min with 1×10^2^ TCID_50_ of each of the three coronaviruses. The virus mixture was then used to infect Vero cells. After 3 days, the cells were fixed and stained with crystal violet to visualize plaques in the confluent cell monolayer, which were subsequently counted. Fig. 2a shows the results of three to four independent experiments, indicating that the infection rate was 50 % of that observed in the absence of the compound at a concentration of 1.73 µg ml^−1^ EGCG (95 % confidence interval (CI) 1.18 to 2.54) for SARS-CoV-2, 4.64 µg ml^−1^ EGCG (95 % CI 2.37–9.29) for MERS-CoV and 0.83 µg ml^−1^ EGCG (95 % CI 0.30–2.41) for SARS-CoV. The green tea compound EC had only a marginal effect on coronavirus infections (IC_50_ >20 µg ml^−1^). The data clearly show that EGCG also has an inhibitory effect on SARS-CoV-2, MERS- and SARS-CoV infection when the replicating virus is used. In addition, similar to Huh7 cells, Vero cells were less sensitive to the residual toxicity of EGCG (Fig. 2b). The cells were only marginally affected at high concentrations of EGCG after 24 or 48 h. Compared to the inhibition of pseudotyped vector entry, slightly lower IC_50_ values were obtained using a virus plaque reduction assay. Nevertheless, the inhibition data obtained with pseudotyped vectors were thus verified with replicating virus.

### EGCG interferes with SARS-CoV-2 receptor binding

**Fig. 3.**
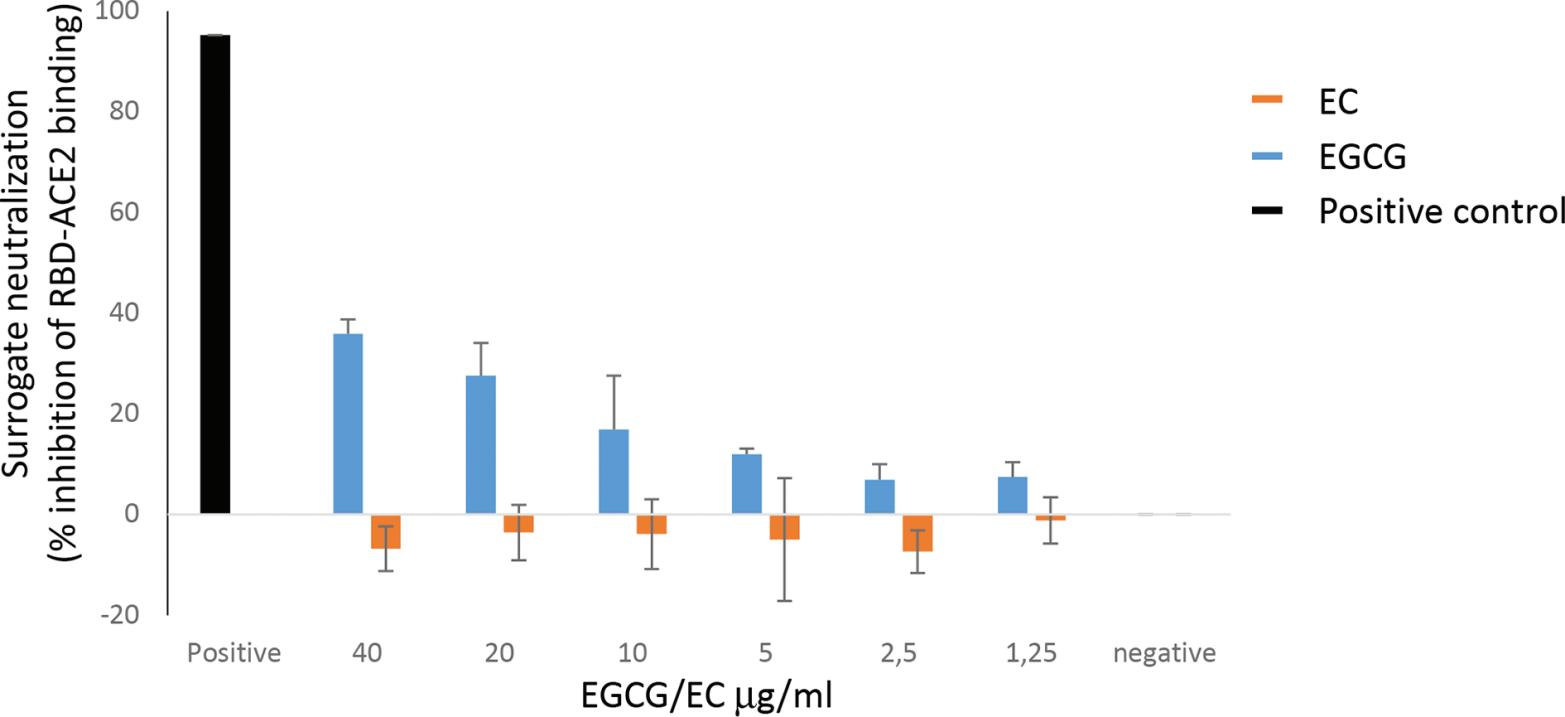
EGCG inhibits attachment of SARS-CoV-2 RBD to ACE2. The sVNT assay was performed with the indicated concentrations of EGCG (black) or EC (grey) according to the manufacturer’s protocol (SARS-CoV-2 sVNT Kit; Genscript, Leiden, Netherlands). Mean values and standard deviations of three independent experiments done in duplicate are given. The negative (non-inhibiting) and positive (inhibiting) controls of the sVNT kit were included. Inhibition was calculated following the manufacturer’s protocol using the following equation: Inhibition in % = (1 – OD of sample/OD of negative control) ×100.

Next, the molecular mechanism of the antiviral activity was investigated. The SARS-CoV-2 RBD, which is located in the S1 protein, interacts strongly with hACE2. The SARS-CoV-2 sVNT Kit is a blocking ELISA that mimics this virus–receptor binding process. The ACE2 protein is coated onto ELISA plates and binding of the horseradish peroxidase-conjugated SARS-CoV-2 RBD is measured as optical density (OD) elicited by a substrate. Neutralizing antibodies or compounds that bind to RBD compete with ACE2 binding and the consequent reduction in OD can be quantified as a surrogate for neutralization. EGCG or EC were applied at different concentrations and only EGCG exhibited a dose-dependent neutralization activity (Fig. 3). Adding EC at the same concentration did not inhibit the ACE2–RBD interaction. The negative (non-inhibiting) and positive (inhibiting, 95 % inhibition) controls of the sVNT kit were included in the assay and performed as expected. This suggests that EGCG, at least partially, interferes directly with SARS-CoV-2 receptor binding.

### EGCG has inhibitory effects when added early during infection

**Fig. 4.**
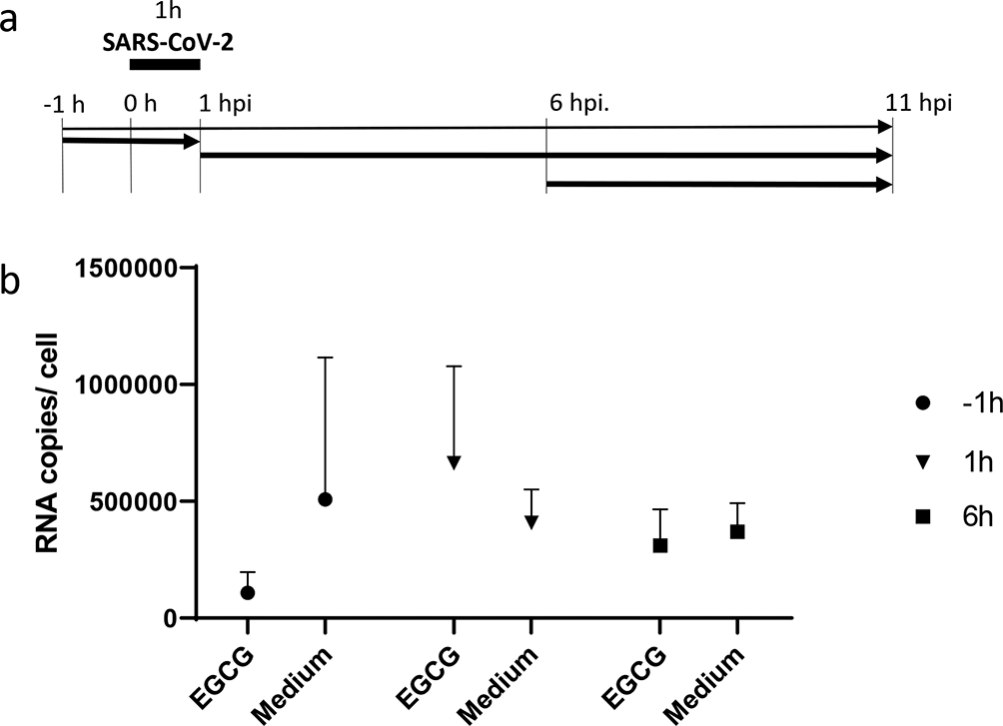
Time-of-drug-addition experiment. (**a**) Vero cells were infected with SARS-CoV-2 and 10 µg ml^−1^ EGCG was added either 1 h before the infection, 1 h after or 6 h after the start of the infection. SARS-CoV-2 was left on the cells for 1 h (black bar), was removed and medium was changed to either medium containing 10 µg ml^−1^ EGCG or medium only. Bold arrows represent incubation with 10 µg ml^−1^ EGCG. After 11 h of infection, cells were lysed and analysed using RT-qPCR. (**b**) Mean values and standard deviation of SARS-CoV-2 –specific RNA copies of three independent experiments at different time points of infection.

The mode of EGCG action on SARS-CoV-2 infections was further evaluated by time-of-drug-addition experiments. Vero cells were infected with SARS-CoV-2 and 10 µg ml^−1^ EGCG was added either 1 h before the infection, 1 h after or 6 h after the start of the infection. SARS-CoV-2 was left on the cells for 1 h and was removed and medium was changed to either medium containing 10 µg ml^−1^ EGCG or medium only (Fig. 4a). After 11 h of infection, cells were lysed and analysed using RT-qPCR. Fig. 4b represents the results obtained as mean values of three independent experiments. Only when EGCG was added 1 h before the infection an inhibitory effect could be observed. While, this effect was not statistically significant, it also indicates that EGCG mainly interferes with early steps in SARS-CoV-2 infection.

## Discussion

In summary, we have shown that the most biologically active compound of green tea extract, EGCG, significantly inhibits SARS-CoV-2 and other coronavirus infections. In contrast, the less biologically active green tea catechin, EC, had no inhibitory effect. This inhibition was also observed with pseudotyped lentiviral vectors. Moreover, EGCG used at concentrations that inhibit the virus did not display toxicity to the target cells. Toxicity of EGCG towards HEK293T-ACE2 cells was much higher than towards Vero and Huh7 cells. This might be caused by the anti-tumour activity of EGCG, which has more effect on these highly transformed cells compared to Vero cells or other cell lines, as has been described before [10, 36, 37]. Correspondingly, binding of SARS-CoV-2 RBD to its receptor ACE2 was inhibited in a surrogate neutralization assay and in time-of-drug-addition experiments, only adding EGCG before the SARS-CoV-2 infection, reduced the infection rate. This indicates that EGCG at least partially blocks the entry of SARS-CoV-2 into the target cells. However, also MERS- and SARS-CoV infections and pseudotyped vectors were inhibited by EGCG, which indicates that a more general effect on virus entry is involved.

EGCG has a broad antiviral effect against many unrelated viruses and has been described to act by competing with heparan sulphate or sialic acid on the target cells for virion binding [9]. It has been described before for other viruses, that EGCG acts directly on the virions and EGCG interacts with viral surface proteins to inhibit the attachment of herpes simplex virus-1, HCV, influenza virus A, vaccinia virus, adenovirus, reovirus, and VSV [9]. These studies showed that EGCG inhibits these unrelated viruses by competing with heparan sulphate or sialic acid for the initial binding of virus particles to cells [9]. Coronaviruses use glycans for the initial attachment to target cell [38]. In addition, it has been described that the SARS-CoV-2 spike protein interacts with cellular heparan sulphate as well as ACE2 through its RBD and heparin blocks the infection of cells by virus and pseudotyped vectors [39]. Therefore it is highly likely that EGCG also inhibits cell attachment and at least for SARS-CoV-2, additionally the RBD-ACE2 interaction. The lower inhibition rate in the *in vitro* sVNT assays compared to the cell based assays might reflect the additional effect of EGCG on interfering with heparin binding.

However, different modes of action have been described for other viruses. EGCG inhibited hepatitis B virus (HBV) by ERK1/2-mediated downregulation of hepatocyte nuclear factor 4α, resulting inhibited HBV gene expression and replication [40, 41]. But effects of EGCG on HBV have also been described to involve enhanced lysosomal acidification, which was unfavourable for HBV replication [42] or having a function as a potent entry inhibitor [43]. Moreover, inhibition of Epstein-Barr virus (EBV) by EGCG has been attributed to MEK/ERK1/2 and PI3-K/Akt pathways, which are involved in the reactivation of EBV into the lytic cycle [44]. But also, a block of binding of EBNA1 to oriP-DNA by EGCG has been described, which inhibited episomal EBV maintenance and transcriptional enhancement [45]. A summary of the pathways involved in EGCG mode of action can be found in Xu *et al*. (2017) [12]. Green tea extracts have been proposed before by *in silico* modelling and in *in vitro* assays as inhibitors of the SARS-CoV-2 chymotrypsin-like protease [46, 47]. However, the IC_50_ values for EGCG mediated inhibition were lower for virus compared to pseudotyped vectors, which suggests that a dominate effect of EGCG on SARS-CoV-2 protease is not very likely.

This broad antiviral effect was also seen here for coronavirus- and VSV-G-pseudotyped vectors, although for VSV-G with slightly less efficacy. Inhibition of VSV or pseudotyped vector entry by EGCG has been described previously and is now confirmed by our results [9, 10].

The IC_50_ of the inhibitory effect observed with SARS-CoV-2 was 1.72 µg ml^−1^. This corresponds to 3.14 μmol, which is within the range commonly described for the antiviral effects of EGCG [11]. However, EGCG might have additional beneficial effects for COVID-19 patients due to its anti-inflammatory effects [48]. Cytokine storm and inflammation are the main causes of severe COVID-19 and anti-inflammatory drugs like dexamethasone have shown promise as a treatment for COVID-19 [48, 49]. However, treatment of SARS-CoV-2 infections by oral tea consumption does not seem to be a realistic perspective. The consumption of two cups of green tea has been reported to result in a peak EGCG plasma level of <1 µM [50]. However, as a small compound with broad antiviral activity, EGCG could potentially be used as a lead structure to further develop highly effective antiviral drugs.
